# Effect of Extract and Ellagic Acid from *Geranium schiedeanum* on the Antioxidant Defense System in An Induced-Necrosis Model

**DOI:** 10.3390/antiox7120178

**Published:** 2018-11-30

**Authors:** Nancy Vargas-Mendoza, Miguel Vázquez-Velasco, Laura González-Torres, Juana Benedí, Francisco José Sánchez-Muniz, Jose Antonio Morales-González, Osmar Antonio Jaramillo-Morales, Carmen Valadez-Vega, Mirandeli Bautista

**Affiliations:** 1 Área Académica de Farmacia, Universidad Autónoma del Estado de Hidalgo, Abasolo N. 600, Colonia Centro, Pachuca, Hidalgo CP 42000, Mexico; nancyv@uaeh.edu.mx (N.V.-M.); osmar_jaramillo@uaeh.edu.mx (O.A.J.-M.); 2 Facultad de Farmacia, Universidad Complutense de Madrid, Ciudad Universitaria, Plaza de Ramón y Cajal S/N, 28040 Madrid, Spain; m.vazquez@ucm.es (M.V.-V.); laura.gonzalez.torres@madrid.org (L.G.-T.); jbenedi@farm.ucm.es (J.B.); frasan@farm.ucm.es (F.J.S.-M.); 3 Instituto Politécnico Nacional, Escuela Superior de Medicina, Plan de San Luis y Díaz Mirón, Col. Casco de Santo Tomás, Del. Miguel Hidalgo, Hidalgo DF 11340, Mexico; jmorales101@yahoo.com.mx; 4 Área Académica de Enfermería, Universidad Autónoma del Estado de Hidalgo, Abasolo N. 600, Colonia Centro, Pachuca, Hidalgo CP 42000, Mexico; 5 Área Académica de Medicina, Universidad Autónoma del Estado de Hidalgo, Abasolo N. 600, Colonia Centro, Pachuca, Hidalgo CP 42000, Mexico; maria_valadez2584@uaeh.edu.mx

**Keywords:** antioxidant activity, *Geranium schiedeanum*, flavonoids hepatoprotective effect, liver damage

## Abstract

*Geranium schiedeanum* has been used in traditional therapies as an antiseptic, antipyretic, and as analgesic. The present study was designed to evaluate the pretreatment with *G. schiedeanum* total extract (GS) and its active metabolites on stimulating the endogenous antioxidant defense system (EADS): catalase (Cat), superoxide dismutase (SOD), glutathione peroxidase (GPx), glutathione reductase (GR), and glutathione reduction index (RI GSH/GSSG) in rat liver treated with a sublethal dose (6.6 mmol/Kg) of thioacetamide (TAA) in order to probe the capacity of GS and the active compounds to reduce liver injury. This was assessed by measuring aspartate aminotransferase (AST), alanine aminotransferase (ALT), and total bilirubin (BILT) in rats pretreated or not with TAA, and pretreated or not with GS and its metabolites. The results showed that GS was able to induce the production of EADS enzymes, increasing redox index GSH/GSSG at 24 and 48 h after intoxication, and both the extract and the ellagic acid exhibited a significant reduction of hepatic damage markers. Our data confirmed the hepatoprotective effect of GS and its metabolites, like ellagic acid, support the possible use of this extract in the treatment of liver injury.

## 1. Introduction

The liver is one of the organs that are most susceptible to damage due to its contact with multiple drugs, environmental toxins, alcohol, etc. This implies a high worldwide incidence of liver diseases. Treatment of these liver diseases is costly in many respects because it involves a series of systemic alterations in addition to the economic expense associated with the disease and its complications. Conjointly, the use of drugs for the treatment of liver disease is frequently not the most indicated, giving rise to drug-induced hepatoxicity, an event that aggravates the pathological condition to an even greater degree. Due to the high incidence of liver diseases, different experimental models have been designed in order to know the molecular, biochemical, and physiopathological mechanisms by which a liver lesion is developed. A frequent model utilized for representing acute damage is a single dose of thioacetoamide (TAA). TAA is considered a potent hepatotoxic that causes damage at the centrilobular level; it is fundamentally metabolized in the CYP2E1, localized in the hepatic microsomal space, which is highly reactive and capable of binding covalently to form adducts with the lysine ε-residues of nuclear proteins, favoring the occurrence of fibrosis, cirrhosis, and cancer [1]. Biochemical disorders in liver tissue induced by TAA have been studied in depth and are highly similar to acute liver lesion in humans, rendering it a reliable model for the evaluation of plant extracts or compounds that appear to delay or return the damage [2].

A lot of plant extracts have been studied in liver-damage models and have proven to be effective by lessening certain markers of the lesion; in this order, the *Geranium* species have also been studied, demonstrating antioxidant [3], anti-inflammatory [4], and antiviral [5] biological activity. For example. in *Geranium sibiricum* L., three main compounds have been identified: geraniin, corilagiin, and gallic acid, of which the first has the highest antioxidant activity, but all three inhibit the xanthine oxidase oxidative stress marker [6] In *Geranium bellum Rose*, a native species of Hidalgo State, Mexico, it has been proposed that the brevifolin carboxylate derivatives identified in this species can be utilized in the treatment of Chagas disease, thanks to the inhibitory effect of the triosephosphate isomerase enzyme of *Trypanosoma cruzi* [7].

**Figure 1:**
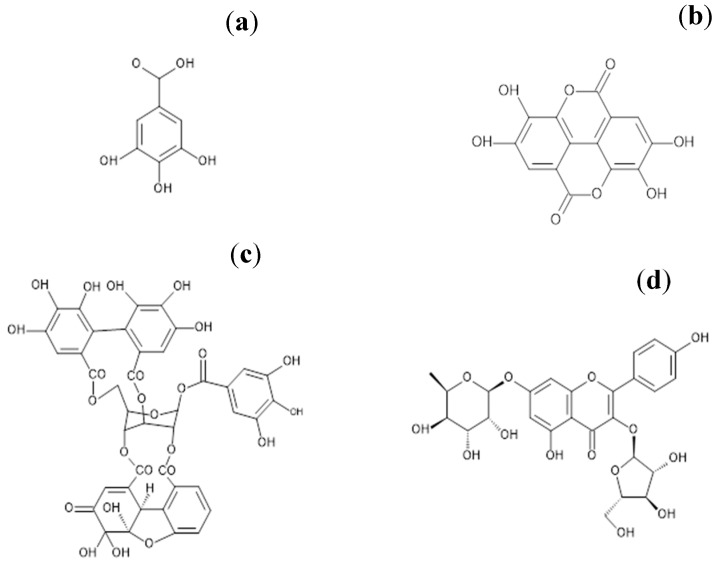
Chemical structure of the active metabolites from *Geranium schiedeanum* extract: (**a**) gallic acid, (**b**) ellagic acid, (**c**) acetonyl geraniin, and flavonoid (**d**) 3-*O*-α-l-arabinofuranoside-7-*O*-β-d-rhamnoside of kaempferol.

On the other hand, *Geranium schiedeanum* belongs to the *Geraniaceae* family, in which there are eight species in Hidalgo, Mexico. Its therapeutic use has been described as a wound antiseptic and as an antipyretic. In a preliminary chemical and pharmacological study performed by our group [8], three hydrolyzable tannins were identified: (a) gallic acid, (b) ellagic acid, (c) acetonile geraniin, and a flavonoid: (d) 3-*O*-α-l-arabinofuranoside-7-*O*-β-d-rhamnoside of kaempferol. Of these, acetonile geraniin was 54% of the extract; it was the component found in the greatest proportion in the extract (Figure 1).

In the TAA-induced hepatotoxicity model in rats, the extract of *G. schiedeanum* proved to diminish and delay liver lesion at levels near those of the control during the first 24 h after intoxication [8,9].

The present study was carried out to investigate the Endogenous Antioxidant Defense System (EADS) in hepatic tissue when compared with the presence of the *G. schiedeanum* extract and the main metabolites in a model where tissue lesion with TAA had been induced.

## 2. Materials and Methods

### 2.1. Preparation of G. schiedeanum Extract

To obtain the extract, we use the method previously described by Gayosso-de-Lucio et al. [8]. This method consisted of extracting 1 kg of the dry and ground aerial parts of *G. schiedeanum* by maceration for seven days with 20 L of acetone water (at a ratio of 7:3) and concentrated by reduced pressure until obtaining a volume of 5 L of aqueous residue. After filtration, the crude extract was concentrated to dryness under vacuum, yielding 112 g of crude extract.

### 2.2. Purification of the Main Active Ingredients of G. schiedeanum

The phytochemical study of the aqueous phase of *Geranium schiedeanum* led to the isolation of some polyphenolic compounds described as: (a) gallic acid, (b) ellagic acid, (c) acetonylgeraniin, and (d) 3-*O*-α-l-arabinofuranoside-7-*O*-β-d-rhamnoside of kaempferol. It is relevant to note that the yield of compound (b) in the crude extract was 54%. Ellagic acid was acquired commercially from Sigma-Aldrich.

Kaempferol 3-*O*-α-l-arabinofuranósyl-7-*O*-α-l-ramnopyranoside (II): Yellow amorphous powder; m.p. 190–192 °C; mg ($\alpha_{D}^{25{}^{\circ}}$ = −168 °C = 0.0098 in MeOH); ^1^H NMR (400 MHz) DMSO-*d*_6_: δ 8.09 (d, 2, *J* = 8.8 Hz, H-2′, H-6′), 6.92 (d, 2, *J* = 8.8 Hz, H-3′, H-5′), 6.84 (d, 1, *J* = 2.0 Hz, H-8), 6.46 (d, 1, *J* = 2.0 Hz, H-6), 5.66 (br, s, 1, H-1″), 5.57 (d, 1, *J* = 1.5 Hz, H-1″′), 4.18 (br, d, 1, *J* = 2.9 Hz, H-2″′), 3.86 (br, s, 1, H-2″), 1.15 (d, 3, *J* = 6.36 Hz, H-6″′) ^13^C NMR (100 MHz) DMSO-*d*_6_: δ 178.9 (C-4), 162.7 (C-7), 161.9 (C-5), 161.2 (C-4′), 158.3 (C-2), 156.9 (C-9), 134.7 (C-3), 131.9 (C-2′, C-6′), 121.6 (C-1′), 116.5 (C-3′, C-5′), 109.1 (C-1″), 106.7 (C-10), 100.4 (C-6), 99.3 (C-1″′), 95.6 (C-8), 87.4 (C-4″), 83.1 (C-2″), 78.1 (C-3″), 72.6 (C-4″′), 71.3 (C-3″′), 71.1 (C-5″′), 70.8 (C-2″′), 61.9 (5″), 18.9 (C-6″′). The spectra obtained in this study were ratified with the data obtained by Gayosso-De-Lucio et al. [7].

### 2.3. Animals and Treatment with the Extract of G. schiedeanum and Active Metabolites

Male Wistar rats aged 10 weeks (weighing 237–265 g) were used in this study. All animals were obtained from the vivarium of the Autonomous University of Hidalgo State. The animals were habituated in polypropylene boxes and fed with commercial feed and water ad libitum with 12/12 h light/dark cycles and temperature control for one week. The experimental protocol consisted of two sets of experimental groups. In the first set, male Wistar rats were randomized into five groups. Groups 3 and 5 were treated for four consecutive days with the extract of *G. schiedeanum* (100 mg/kg) intragastrically (i.g.). On day 4, Group 2 and 5 animals were administered one dose per 6.6 mmol/kg of TAA dissolved in 1 mL NaCl (9%) intraperitoneally (i.p.). At 24 h after TAA administration, Groups 2 and 3 were sacrificed, and at 48 h, Groups 1, 4, and 5 were sacrificed. In the second set, the animals were divided into six groups: (1) Control; (2) TAA; (3) Extract + TAA; (4) Geraniin + TAA; (5) Kaempferol + TAA, and (6) Ellagic acid. Group 1 did not receive any type of treatment, Group 2 received a unique dose of TAA (6.6 mmol/g) via the i.p. route, and Groups 3, 4, 5, and 6 received a four day pretreatment with the extract of *G. schiedeanum*, geraniin, kaempferol, and ellagic acid, respectively (100, 25, 2.5, and 12.5 mg/kg) via the i.g. route, and, on day 4, were administrated a unique dose of TAA (6.6 mmol/g) i.p.

The experiment was carried out in duplicate in four animals for each condition following international and national criteria for the use and care of animals for experimentation, as described in The Guiding Principles in the Use of Animals in Toxicology, adopted by the Society of Toxicology in 1989 and the Official Mexican Norm (NOM 062-ZOO-1999).

### 2.4. Determination of Antioxidant Enzymes by Western Blot

Determination of EADS enzymes was conducted by means of the Western blot technique, in which the enzymes were submitted to electrophoresis (Mini Protean 3 Electrophoresis System and, as power source, Power Pac High-Current (all these purchased from Bio-Rad, Hercules, CA, USA)), in acrylamide gel, and later immunotransference based on the specificity of antibody–antigen recognition. Equal amounts of protein lysates obtained from rat-liver homogenates were separated in 10% sodium dodecyl sulfate–polyacrylamide (SDS–PAGE) gel electrophoresis. The gels were blotted onto a polyvinylidene difluoride (PVDF) Amersham Hybond-P membrane (GE Healthcare, Buckinghamshire, UK) and incubated with the appropriate antibodies: antisuperoxide dismutase (S2147; 1:2000 dilution), anticatalase (C0979; 1:3000 dilution), anti-β-actin (A2228; 1:50,000 dilution) from Sigma-Aldrich, St. Louis, MO, USA; antiglutathione peroxidase (Ab59546; 1:5000 dilution) from Abcam, Cambridge, UK; antiglutathione reductase (sc-32886; 1:500 dilution), mouse antigoat (sc-2490; 1:20,000 dilution) and goat antirabbit (sc-2004; 1:10,000 dilution) from Santa Cruz Biotechnology, Dallas, TX, USA). Blots were developed by enhanced chemoluminescence using an Amersham ECL Plus Western Blotting Detection Reagents (GE Healthcare, Buckinghamshire, UK) according to the manufacturer’s instructions. β-actin was used as a loading control [10].

### 2.5. Determination of Reduced Glutathione/Oxidized Glutathione (GSH/GSSG)

Determination of glutathione was performed by fluorescence from rat-liver homogenate; for GSH quantification, a fluorescent *O*-phthaladehyde catheter was utilized (Sigma-Aldrich) and a buffered-phosphate solution of ethylenediaminetetraacetic acid (EDTA) was employed, while for GSSG, a solution of *N*-ethylmaleimide (Sigma-Aldrich) was utilized. This was mixed and incubated at room temperature for 15 min in the dark. Fluorescence was at λ_exc_ = 340 nm and λ_em_ = 420 nm. The reduction index (IR) was calculated (GSH/(GSH + GSSG)) for each sample.

### 2.6. Sample Processing

All the animals were sacrificed at 24 h; an opening was made in the abdominal cavity and portal blood samples were obtained in heparinized tubes, which were centrifuged at 4000 rpm for 15 min at 16 °C to obtain the serum in Eppendorf tubes, which were stored under ultrafreezing conditions at −80 °C until requirement. Additionally, the liver was extracted, which was cut into small fragments, wrapped in aluminum foil, and placed in plastic bags for storage under ultrafreezing conditions at −80 °C to preserve its integrity.

### 2.7. Determination in Serum of Aspartate Aminotransferase (AST), Alanine Aminotransferase (ALT), and Total Bilirubin (BILT)

Quantitative determination of the AST enzyme was carried out with the GOT (AST)-LQ SPINREACT kit by the method of Rej and Horder [11]. The activity of this enzyme was measured by the diminution in absorbance to 340 nm at 25 °C, produced by the oxidation of NADH into NAD^+^ in the paired reaction of the reduction of oxaloacetate into malate, catalyzed by malate dehydrogenase. Quantitative determination of the ALT enzyme was performed with the GTP (ALT)-LQ SPINREACT kit by the method of Murray [12]. The activity of this enzyme was measured by diminution in absorbance to 340 nm at 25 °C, produced by the oxidation of NADH into NAD^+^ in the coupled reaction of the reduction of pyruvate into lactate, catalyzed by lactate dehydrogenase.

Quantitative determination of BILT was performed with the BILIRUBIN T-DMSO SPINREACT kit. The previously described method [13] is based on the conversion of bilirubin into azobilirubin by means of diazotized sulfanilic acid; measuring this at 555 nm at 25 °C, the color intensity formed was proportional to the concentration of bilirubin present in the sample. To the determination of indirect bilirubin, direct bilirubin was also determined, corresponding to the result at total bilirubin.

### 2.8. Statistical Analysis

All data were expressed as the mean ± standard deviation (±SD) submitted to one-tailed analysis of vriance (ANOVA) in four animals for each condition in a variable number of experiments specified for each case. Analysis for evaluating significant differences between groups was carried out with the Tukey test. Significant differences were considered at a value of *p* < 0.05. Data were processed with the SPSS version 15.0 statistical software package (SPSS, Inc., Chicago, IL, USA).

## 3. Results and Discussion

Alternative therapies on pathologies of the liver continue to be scarcely explored area; so, it is timely to present proposals of novel treatments that would be less costly and that would imply a smaller number of molecular alterations at distinct levels. Therefore, it is of great importance that the correct use of plants as part of complementary alternative medicine (CAM) be based on phytochemical and biological studies that permit an explanation of the interaction phenomena between plant compounds and physiological responses in live systems.

### 3.1. Effect of Pretreatment with G. schiedeanum Extract on EADS Enzymes in Rat Liver after Intoxication with TAA

**Figure 2:**
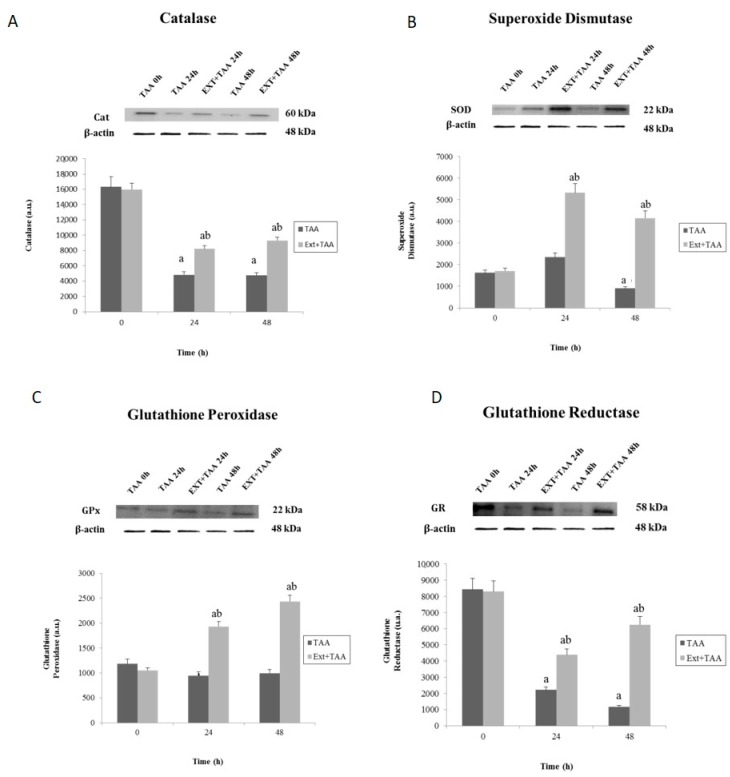
Effect of *G. schiedeanum* extract pretreatment on levels of (**A**) catalase (Cat), (**B**) superoxide dismutase (SOD), (**C**) glutathione peroxidase (GPx), and (**D**) gluatathione reductase (GR) analyzed by Western blot in liver homogenates of rats intoxicated with a sublethal dose of thioacetamide (TAA). Samples were obtained at 0, 24, 48 h after the administration of the TAA. The results expressed in arbitrary units (a.u.) are the mean SD ± of two determinations from four different animals for each condition. The differences compared to the respective controls are represented as (a) and the differences due to extract are expressed as (b), *p* < 0.05.

The EADS is the product of a long evolution in the face of the incessant damage that living beings have faced in the environment. As its name implies, this system functions as a protective against the toxic agents generating oxidative stress (OS) in the metabolic response. OS is defined as the disequilibrium between the production of reactive oxygen species (ROS), and the antioxidant defenses against these molecules. Consequently, this system undergoes fluctuations proportional to the dose and time of contact with the toxin, and it is precisely these variations that, on being measured, offer us a schema regarding the damage caused and, in turn, of the capacity of the selfsame organism to recover. For the purpose of this investigation, the effect of pretreatment with the extract of *G. schiedeanum* was evaluated on the levels of some of the most important enzymes constituting EADS (catalase (Cat), superoxide dismutase (SOD), glutathione peroxidase (GPx), and glutathione reductase (GR)) in the liver of rats intoxicated with a sublethal dose of TAA. One of the most important systems of cellular protection against the toxic effects of molecular oxygen is that which the SOD and Cat enzymes carry out in conjunction. The SOD enzyme is charged with catalyzing the dismutation of O_2_^−•^ that, in turn, generates H_2_O_2_; this substance is transformed into H_2_O by Cat. It is known that the modulatory action that these enzymes possess to protect each other when O_2_^−•^ is produced is inactivated by Cat, and the production of H_2_O_2_ inhibits SOD [14]. The results indicated alterations at distinct levels on the concentration of these enzymes in liver tissue. Catalase underwent a significant decrease at 24 h in response to the OS generated by intoxication with TAA. However, the group that received the extract raised the level of catalase, which increase, though it did not reach baseline levels, was significant; at 48 h, the value decreased even more in animals treated with TAA, while the group of the extract attempted to approximate the controls (Figure 2A). SOD, on its part, presents certainly distinct behavior: on initiation, the values of the controls became somewhat low and, after the first 24 h passed, these increase slightly in the group injected with TAA. In contrast, the group that received the extract exhibited a significant increase in the SOD value. At 48 h, in the group with TAA, the SOD level decreased even more, and the group pretreated with the extract similarly slightly diminished its SOD value, while the difference continued to be extraordinarily significant (Figure 2B).

On the other hand, GPx presented slightly reduced values at 24 h of intoxication with TAA, but the group that received the extract considerably increased its values, and the rise was even more pronounced at 48 h (Figure 2C). As for the GR enzyme, high values that were observed at the beginning (at 0 h) declined at 24 and 48 h, both for the TAA group, as they did for the Ext + TAA group, which showed significant difference compared to its respective controls. On the other hand, significant differences were observed between groups (TAA and Ext + TAA) at 24 and 48 h (Figure 2D).

Moreover, GPx, GR, and GSH define activities in conjunction. GPx catalyzes the reaction of the oxidation of GSH, converting it into GSSG, taking H_2_O_2_ or other organic peroxides as substrate. In parallel, GR participates in the restoration of glutathione to its reduced form, GSH, with the help of a reducer agent; in this case, thanks to the presence of NADPH due to the action of G6P dehydrogenase that, in turn, is provided through the pentose phosphate pathway. Taken together, all of these components protect from the constant deterioration to which cellular systems are exposed.

Currently, there are not enough studies to explain the molecular mechanisms that help to better understand the effects observed in scientific experimentation between metabolites of natural products and cellular systems. Specifically, the phenomena of genetically expressed antioxidant enzymes are considered processes that are difficult to elucidate due to the enormous genetic variation among individuals, and environmental factors. Notwithstanding this, in this study, we were able to observe the different responses in each of the EADS enzymes. The results suggest that the extract of *G. schiedeanum* works perfectly as a liver protective when damage has been induced by exposure to a highly toxic agent (TAA) since, when the extract was administered, the totality of the enzymes, at distinct levels, increased their concentration significantly in liver tissue.

### 3.2. Effect of Pretreatment with the Extract of G. schiedeanum on the GSH/GSSG Reduction Index (RI) in TAA-Intoxicated Rats

**Figure 3:**
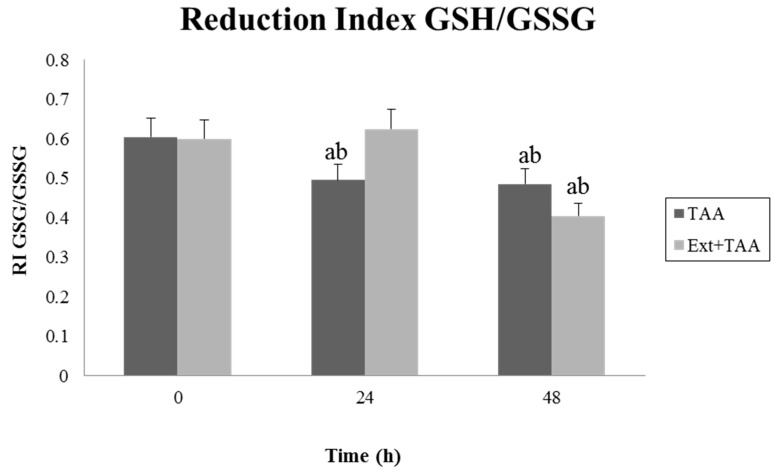
Effect of *G. schiedeanum* extract pretreatment in the Reduction Index (RI) reduced glutathione/oxidized (GSH/GSSG) analyzed by measuring fluorescence in liver homogenates of rats intoxicated with a sublethal dose of TAA. Samples were taken at 0, 24, and 48 h after the administration of the TAA. The results were expressed as the mean ± SD of duplicate values obtained in liver homogenates from four different animals for each condition. The differences compared to the respective controls are represented as (a) and the differences due to extract are expressed as (b), *p* < 0.05.

Evaluation of the GSH/GSSG redox index in homogenates of rat liver by the effect of TAA intoxication exhibited the evident reduction of GSH at 24 and 48 h; contrariwise, pretreatment with the extract of *G. schiedeanum* demonstrated recovery of the redox index on increasing the concentration of GSH, even above that of the control group, at 24 h after receiving the hepatotoxic. The same effect was not observed in the 48-h group that was also treated with the extract (Figure 3).

Very probably, the sulfdioxide (TASO_2)_ metabolite, which is highly electrophilic, interacts with the hepatic GSH, forming conjugates by means of the action of GST, to afterward be hydrolyzed in each of their parts and eliminated through the bile; this phenomenon is appreciated by the diminution of GSH in our experiment. Lesser depletion of hepatic GSH is possibly due to molecular interaction among the antioxidant compounds contained in the extract, with TASO_2_ and ROS generated during intoxication with TAA. The most abundant nonprotein thiolic glutathione compound in the cells is constituted of the amino acids l-glutamine, l-cysteine, and l-glycine; it has been associated with different physiological processes, among which the following are prominent: elimination of xenobiotics, cellular signaling, and as antioxidant and reducer agent on interacting and neutralizing electrophilic compounds and ROS. It is essential to maintain the redox index of glutathione reduced above the oxidase (GSH/GSSG), which is elevated in cellular systems because diminution of this index implies an increase in GSSG production and a diminution of the thiol groups linked with protein destabilization due to the formation of mixed disulfate outcomes, lipid peroxidation, protuberances in the cellular membrane, an increase in intracellular calcium, and induction of apoptosis by caspase activation due to the opening of the mitochondrial permeability transition pores implicated in the cellular cycle, proliferation, and apoptosis [15]. It is known that the biosynthesis of GSH basically depends on two factors: the bioavailability of cysteine, and the presence of γ-glutamyl cysteine synthetase (γ-GCS). Factors such as a deficiency in methionine diet, from which cysteine is formed, abnormalities of methionine metabolism [16,17], or alterations in the expression of γ-GCS can delay the biosynthesis of the tripeptide [4,18], which is not necessarily linked with the presence or activity of the GPx or GR enzymes. Thus, it is suggested that perhaps the recovery time of GSH would be greater due to other situations, independent of the presence of EADS enzymes.

### 3.3. Effect of Pretreatment G. schiedeanum Extract and Active Metabolites in liver Damage

**Figure 4:**
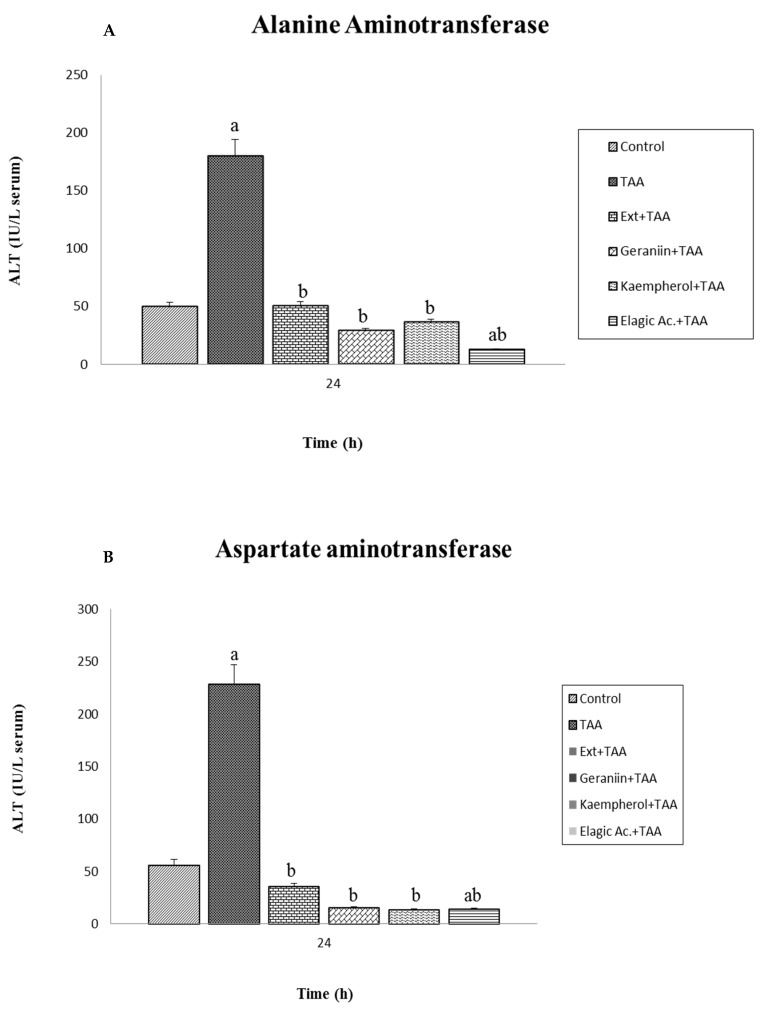
Effect of *G. schiedeanum* extract and active compounds on levels of (**A**) alanine aminotransferase (ALT) and (**B**) aspartate aminotransferase (AST) in serum of rats intoxicated with a sublethal dose of TAA. Samples were obtained at 24 h after intoxication with TAA. The results, expressed in international units per liter of serum (IU/L serum), were the mean ± SD of three measurements for each sample (*n* = 5). The differences compared to the respective controls are represented as (a) and the differences due to extract or compounds are expressed as (b), *p* < 0.05.

The hepatoprotective effect was evaluated in the total extract of *G. schiedeanum* and its main active ingredients in a liver-damage model induced by intoxication with a single dose of TAA in rats with the measurements of clearly known AST, ALT, and BILT enzyme lesion markers. A significant increase was observed (*p* < 0.001) in the group that received the TAA vs. the control group, which confirms the damage produced by acute exposure to TAA. This compound is one of the most frequently studied hepatotoxic in order to induce fibrosis, cirrhosis, and cancer in experimental models with rats. At high doses of 500 mg/kg, the compound generates perivenous hepatic necrosis, the product of acute intoxication [19]. In this experiment, pretreatment with the extract of *G. schiedeanum* reduced AST and ALT levels, with levels near the controls and, in the case of the active compounds, the levels fell even further than the control group (Figure 4A,B).

**Figure 5:**
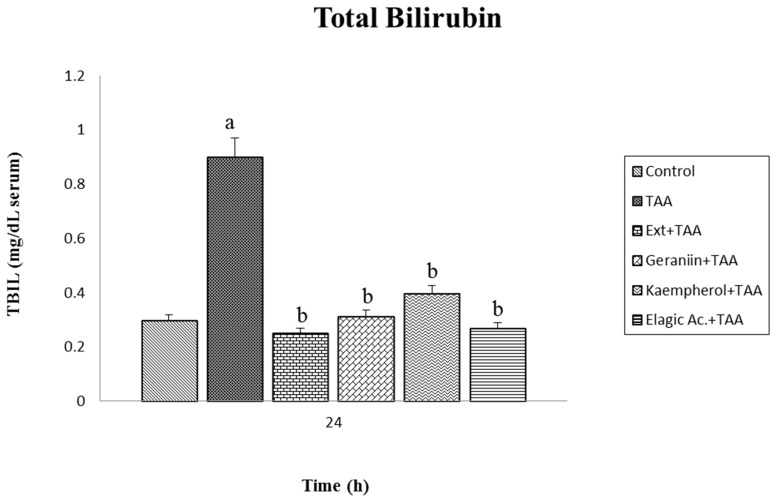
Effect of *G. schiedeanum* extract and active compounds on levels of total bilirrubin (BILT) in serum of rats intoxicated with a sublethal dose of TAA. Samples were obtained at 24 h after intoxication with TAA. The results expressed in milligrams per deciliter of serum (mg/Dl serum) were the mean ± SD of three measurements for each sample (*n* = 5). The differences compared to the respective controls are represented as (a) and the differences due to extract or compounds are expressed as (b), *p* < 0.05.

According to the obtained results, it was observed that ellagic acid (active metabolite) was the compound with the greatest significant reduction (*p* < 0.05) in AST and ALT levels in comparison with geraniin and kaempferol. BILT values in serum undoubtedly increased in animals sacrificed 24 h after receiving the toxic compound; however, the levels reached continued to be considered normal because, according to the references, maximal damage is observed up to 48 h after intoxication (Figure 5). The groups pretreated with the extract of *G. schiedeanum* and its metabolites underwent diminution in the concentration of BILT, with very similar results to those of the control (*p* < 0.05); a statistically significant difference was not identified in the lesion’s capacity of diminution between the extract and the main active ingredients of the plant.

Bioavailability is a determining factor for the evaluated compounds in order for them to achieve the desired effect, and apparently, the combined use of flavonoids increases bioavailability by means of the interactions carried out in transport [12]. There is limited information on the process of biotransformation undergone by the flavonoids in humans. Because of the study of some flavonoids in human microsomes, it is known that these are metabolized by CYP1A2 and CYP2C9 [20]; in particular, kaempferol is transformed into quercitrin by CYP1A1 [21]. The speed with which these are oxidized depends on the degree of methylation that they present, that is, the compounds found to be partially methylated are oxidized more rapidly than those that are completely methylated [6].

Information on ellagitannin bioavailability and metabolism continues to be limited; thus, these processes are not completely understood but, despite this, the study on metabolites found in serum, urine, and in rat feces from the administration of geraniin and corilagin isolated from *Geranium thunbergii*, a species utilized as an antidiarrheic in Japan, indicates high antioxidant activity with the 2,2-diphenyl-1-picrylhydrazyl (DPPH) and the oxygen radical absorbance capacity (ORAC) methods. Even with the latter of these methods, greater activity is observed in the metabolites than in the intact compounds; analysis in serum indicated an increase in antioxidant activity in ORAC on the increase of plasma levels after oral administration of geraniin, such findings suggesting that ellagitannin metabolites can beneficially contribute to health, acting as antioxidants [22]. This information coincides with the results found in our study, proposing that the active ingredients of the extract, acetonile geraniin, 3-*O*-α-l-arabinofuranoside-7-*O*-β-d-rhamnoside of kaempferol, and ellagic acid follow similar kinetic absorbance at the intestinal level and probably in the colon due to the action of the microflora, with relatively rapid absorbance times of between 1 and 2 h. In particular, the ellagitannins (acetonile geraniin and ellagic acid) are transformed into urolithins by intestinal microflora in order to be absorbed [23,24], and the flavonoid can be hydrolyzed by the phlorizin hydrolase for its release from arabinofuranose and ramnose sugars, becoming more lipophilic and similar to the membrane of the intestinal epithelial cells to be absorbed, or also by the action of cytosolic β-glycosidase through the sodium-dependent cellular receptor (GLUT-1) as a second option [5]. Once in systemic circulation, these are metabolized by the action of sulfatases, UDP-glucuronosyltransferases, and catechol-*O*-methyltransferases, these later submitted to phase-II reactions in the liver. On being metabolized in the liver, it is suggested that these metabolites are responsible for directly acting to diminish the OS caused by the TASO_2_ metabolite, the product of the hepatic biotransformation of TAA, which is highly reactive and capable of adhering to subcellular substrates, giving rise to a liver lesion [25]. This is reflected in the significant diminution of damage markers ALT and AST for the group receiving the extract. It is also known that some polyphenols, such as transresveratrol and tannic acid, can modulate the CYP2E1 intervening in the transformation of xenobiotics, reducing their toxicity [26].

A study conducted by Qureshi in 2009 [19] with the extract of *Cordia macleodii* leaves in the liver-damage model induced by Carbon tetrachloride (CCl_4_) demonstrated a significant reduction (*p* < 0.001) of AST, ALT, ALP, and BILT levels; such an effect was attributed to the content of flavonoids and triterpenoids, in which antioxidant [27,28] as well as hepatoprotective [29] activity was found.

In another experiment performed with the aqueous and methanolic extract of *Phyllantus niruri*, also utilizing CCl_4_ as hepatotoxic agent, a hepatoprotective effect was observed on diminishing ALT and AST levels (*p* < 0.05) [30]. Phytochemical studies of this plant report the presence of phyllanthins and hypophyllanthins, which can act against the cytotoxic effect caused by the CCl_4_ [21]. A more recent study [31] with the same plant species demonstrated its ability to considerably reduce the damage caused in rats intoxicated by TAA; the reduction was reported of ALT, AST, ALP, and BILT levels on comparing the results with the control and silymarin, a substance isolated from the leaves of *Silybum marinum*, whose hepatoprotective effect has previously been studied. The increase in the activity of ALT and AST enzymes in intoxicated rats compared with the controls indicated the presence of hepatocyte necrosis, resulting in the deficiency of transaminase and the constant release of ALP, giving rise to cholestasis, which may probably be attributed to structural alterations and to hepatocyte integrity.

The oxidative damage cause by toxic compounds such as CCl_4_ harm the hepatocytes’ plasma membrane; this causes the AST and ALT enzymes, normally found in cytosol, to be released into the bloodstream. Treatment with Picroliv, curcumin, and ellagic acid diminishes the damage caused by CCl_4_, with values near those of the control. The protective effect presents due to preservation in cellular integrity because of the stabilization of the plasma membrane [32].

The experimental results reveal the greatest effect on the reduction of these ALT and AST levels by the total extract and ellagic acid; this is the sole compound that is significantly different from the extract. The reason is probably due to the effect of the hydrolysis that geraniin undergoes due to the microflora releasing the two molecules of ellagic acid that are found bound to its structure; this perhaps implies a greater amount of time, while the ellagic acid is directly transformed into urolithins to enter into the bloodstream; thus, the time is less, possibly exerting an influence on the effect achieved by the compound.

On the other hand, it is well known that the liver possesses the capacity to regenerate in response to damage with different agents, such as ethanol (EtOH), acetaminophen, carbon tetrachloride (CCl4), and thiacetamide; this regeneration is a complex process and it is controlled by diverse intra- and extrahepatic factors [33]. In this regard, some authors propose that OS also participate in liver regeneration, which depends on experimental conditions that could inhibit or favor it. Moreover, it has been found that the nuclear factor erythroid 2-related factor (Nrf-2), a redox-sensitive transcriptional factor, is one of the factors in the regulation of various genes of antioxidant and cytoprotector proteins [34,35]. In this respect, Morales et al. [33] postulate that, because Nrf-2 is a modulator of the enzymes that regulate OS, it should also surely participate in the liver proliferation process. In this way, recent studies report an increase of Nrf-2 levels provided by curcumin in a liver-damage model with EtOH [36]. Moreover, Morales et al. [9] found an increase of Nrf-2 during liver regeneration and the administration of EtOH and/or the *G. schiedeanum* extract, concluding that *G. schiedeanum* exerted an antioxidant effect, diminishing ROS and increasing Nrf-2 expression, favoring liver proliferation.

The use of medicinal plants is accepted in all parts of the world thanks to ancestral knowledge on their health benefits, whether as a preventive or a therapeutic measure in a number of pathologies. However, pertinent phytochemical and biological studies are required to validate their utilization, recognizing the lethal doses that, accompanied by safety measures, allow for their implementation in medical treatments. In Mexico, despite colossal floral variety and the millennial inheritance of pre-Columbian cultures, the employment of medicinal plants has been relegated in a popular and informal manner to a certain social stratum solely as an economic and accessible alternative that has not yet merited sufficient interest to be formally studied.

The importance of the study carried out lies in making a proposal on the use of the *G. schiedeanum* species and its active metabolite as an alternative to diminish liver damage originating from exposure to toxic substances during certain periods and doses that would, in time, probably terminate in some acute disease. In our study, the extract, at a prudent dose, favored the levels of EADS enzymes, including Cat, SOD, GPx, and GR, as well as IR GSH/GSSG, in homogenates of rat liver. In the second part, the assay with the extract proved to significantly reduce AST and ALT damage markers in serum, noting a greater difference in the main active compounds (ellagic acid) than in the extract. It is noteworthy that the mentioned effects were obtained at very low doses of the extract with respect to the estimated LD_50_.

## 4. Conclusions

In this manner, this study provides the first evidence showing that this species possesses high antioxidant and hepatoprotective activity, probably attributed to the content of tannins and flavonoids isolated from the species. This suggests that ellagic acid isolated from *G. schiedeanum* and the total extract may be a good candidate for the treatment of liver injury. Likewise, these results give great importance to the use of plants as part of CAM to treat liver diseases. Therefore, to establish the proper use of this extract in the clinic, it is important to consider these results and formulate appropriate clinical studies to establish the adequate dose in patients. Our results agree with previous studies where it was shown that the extract of *G. schiedeanum* demonstrates antioxidant and hepatoprotective capacity in other liver-injury models as well.

## References

[1] Chilakapati J., Korrapati M.C., Hill R.A., Warbritton A., Latendresse J.R., Mehendale H.M. (2007). Toxicokinetics and toxicity of thioacetamide sulfoxide: A metabolite of thioacetamide. Toxicology.

[2] Wong W.L., Abdulla M.A., Chua K.H., Kuppusamy U.R., Tan Y.S., Sabaratnam V. (2012). Hepatoprotective Effects of *Panus giganteus* (Berk.) Corner against Thioacetamide-(TAA-) Induced Liver Injury in Rats. Evid. Based Complement. Altern. Med..

[3] Miliauskas G., Mulder E., Linssen J.P., Houben J.H., van Beek T.A., Venskutonis P.R. (2007). Evaluation of antioxidative properties of Geranium macrorrhizum and Potentilla fruticosa extracts in Dutch style fermented sausages. Meat Sci..

[4] Miguel O.G., Calixto J.B., Santos A.R., Messana I., Ferrari F., Cechinel Filho V., Pizzolatti M.G., Yunes R.A. (1996). Chemical and preliminary analgesic evaluation of geraniin and furosin isolated from *Phyllanthus sellowianus*. Planta Med..

[5] Toshkova R., Nikolova N., Ivanova E., Ivancheva S., Serkedjieva J. (2004). In vitro investigation on the effect of a plant preparation with antiviral activity on the functions of mice phagocyte cells. Die Pharm..

[6] Shim J.U., Oh P.S., Lim K.T. (2009). Anti-inflammatory activity of ethanol extract from *Geranium sibiricum* Linne. J. Ethnopharmacol..

[7] Gayosso-De-Lucio J., Torres-Valencia M., Rojo-Domínguez A., Nájera-Peña H., Aguirre-López B., Salas-Pacheco J., Avitia-Domínguez C., Téllez-Valencia A. (2009). Selective inactivation of triosephosphate isomerase from *Trypanosoma cruzi* by brevifolin carboxylate derivatives isolated from *Geranium bellum* Rose. Bioorg. Med. Chem. Lett..

[8] Gayosso-De-Lucio J., Bautista M., Velazquez-Gonzalez C., De la O Arciniega M., Morales-Gonzalez J.A., Benedi J. (2014). Chemical composition and hepatotoxic effect of *Geranium schiedeanum* in a thioacetamide-induced liver injury model. Pharmacogn. Mag..

[9] Morales-González A., Bautista M., Mandrigal-Santillán E., Posadas-Mondragón A., Anguiano-Robledo L., Mandrigal-Bajaidar E., Álvarez-González I., Fregoso-Aguilar T., Gayosso-Islas E., Sánchez-Moreno C. (2017). Nrf2 modulates cell proliferation and antioxidants defenses during liver regeneration induced by partial hepatectomy. Int. J. Clin. Exp. Pathol..

[10] Vázquez-Velasco M., González-Torres L., López-Gasco P., Bastida S., Benedí J., Sánchez-Reus M.I., González-Muñoz M.J., Sánchez-Muniz F.J. (2014). Liver oxidation and inflammation in Fa/Fa rats fed glucomannan/spirulina-surimi. Food Chem..

[11] Rej R., Horder M. (1984). Aspartate aminotransferase. l-aspartate: 2-oxoglutarate aminotranferase, EC 2.6.2.1. Routine U.V. method. Methods of Enzymatic Analysis.

[12] Murray R., Kaplan A., Pesce A.J. (1984). Alanine aminotransferase. Clinical Chemistry: Theory, Analysis, and Correlation.

[13] Martinek R.G. (1966). Improved micro-method for determination of serum bilirubin. Clin. Chim. Acta.

[14] Li S., Tan H.Y., Wang N., Zhang Z.J., Lao L., Wong C.W., Feng Y. (2015). The role of oxidative Stress and antioxidants in liver diseases. Int. J. Mol. Sci..

[15] Lu S.C. (2009). Regulation of glutathione synthesis. Mol. Aspects Med..

[16] Mari M., Morales A., Colell A., Garcia-Ruiz C., Fernandez-Checa J.C. (2009). Mitochondrial glutathione, a key survival antioxidant. Antioxid. Redox. Signal.

[17] Mato J.M., Corrales F.J., Lu S.C., Avila M.A. (2002). S-Adenosylmethionine: A control switch that regulates liver function. FASEB J..

[18] Mikstacka R., Gnojkowski J., Baer-Dubowska W. (2002). Effect of natural phenols on the catalytic activity of cytochrome P450 2E1. Acta Biochim. Pol..

[19] Qureshi N., Kuchekar B., Logade N., Haleem M. (2009). Antioxidant and hepatoprotective activity of *Cordia macleodii* Leaves. Saudi Pharm. J..

[20] Rodrigues G.R., Naso D., Cangeri F., Porawski M., Marcolin É., Kretzmann N.A., Ferraz A.D., Richter M.F., Marroni C.A., Marroni N.P. (2012). Treatment with aqueous extract from *Croton cajucara* Benth reduces hepatic oxidative stress in streptozotocin-diabetic rats. J. Biomed. Biotechnol..

[21] Seeram N.P., Henning S.M., Zhang Y., Suchard M., Li Z., Heber D. (2006). Pomegranate juice ellagitannin metabolites are present in human plasma and some persist in urine for up to 48 hours. J. Nutr..

[22] Silva I.D., Rodrigues A.S., Gaspar J., Maia R., Laires A., Rueff J. (1997). Involvement of rat cytochrome 1A1 in the biotransformation of kaempferol to quercetin: Relevance to the genotoxicity of kaempferol. Mutagenesis.

[23] Syamasundar K.V., Singh B., Thakur R.S., Husain A., Kiso Y., Hikino H. (1985). Antihepatotoxic principles of Phyllanthus niruri herbs. J. Ethnopharmacol..

[24] Tatsimo S.J., de Dieu Tamokou J., Havyarimana L., Csupor D., Forgo P., Hohmann J., Kuiate J.R., Tane P. (2012). Antimicrobial and antioxidant activity of kaempferol rhamnoside derivatives from *Bryophyllum pinnatum*. BMC Res. Notes.

[25] Pérez Escandón B.E., Villavicencio Nieto M.Á., Ramírez Aguirre A. (2003). Lista de las Plantas útiles del Estado de Hidalgo.

[26] Walle U.K., Walle T. (2007). Bioavailable flavonoids: Cytochrome P450-mediated metabolism of methoxyflavones. Drug Metab. Dispos..

[27] Brand-Williams W., Cuvelier M.E., Berset C. (1995). Use of a free radical method to evaluate antioxidant activity. LWT Food Sci. Technol..

[28] Zhao B., Waterman M.R. (2011). Moonlighting cytochrome P450 monooxygenases. IUBMB Life.

[29] Aniya Y., Koyama T., Miyagi C., Miyahira M., Inomata C., Kinoshita S., Ichiba T. (2005). Free radical scavenging and hepatoprotective actions of the medicinal herb, *Crassocephalum crepidioides* from the Okinawa Islands. Biol. Pharm. Bull..

[30] Harish R., Shivanandappa T. (2006). Antioidant activity and hepatoprotective potential of *Phyllantus niruri*. Food Chem..

[31] Amin Z.A., Bilgen M., Alshawsh M.A., Ali H.M., Hadi A.H., Abdulla M.A. (2012). Protective Role of Phyllanthus niruri Extract against Thioacetamide-Induced Liver Cirrhosis in Rat Model. Evid. Based Complement. Altern. Med..

[32] Girish C., Pradhan S.C. (2012). Hepatoprotective activities of picroliv, curcumin, and ellagic acid compared to silymarin on carbon-tetrachloride-induced liver toxicity in mice. J. Pharmacol. Pharmacother..

[33] Morales-González J.A., Mandrigal-Santillán E., Morales-González A., Bautista M., Gayosso-Islas E., Sánchez-Moreno C. (2015). What is Known Regarding the Participation of Factor Nrf-2 in Liver Regeneration?. Cells.

[34] Keum Y.S., Choi B.Y. (2014). Molecular and chemical regulation of the Keap1-Nrf2 signaling pathway. Molecules.

[35] Han X., Shuen T., Lou H. (2007). Dietary poliphenols and their biological significance. Int. J. Mol. Sci..

[36] Xiong Z.E., Dong W.G., Wang B.Y., Tong Q.Y., Li Z.Y. (2015). Curcumin attenuates chronic ethanol-induced liver injury by inhibition of oxidative stress via mitogen-activated protein kinase/nuclear factor E2-related factor 2 pathway in mice. Pharmacogn. Mag..

